# Tablet-Based Strength-Balance Training to Motivate and Improve Adherence to Exercise in Independently Living Older People: A Phase II Preclinical Exploratory Trial

**DOI:** 10.2196/jmir.2579

**Published:** 2013-08-12

**Authors:** Patrícia Silveira, Rolf van de Langenberg, Eva van het Reve, Florian Daniel, Fabio Casati, Eling D de Bruin

**Affiliations:** ^1^University of TrentoDepartment of Information Engineering and Computer ScienceTrentoItaly; ^2^Institute of Human Movement Sciences and SportDepartment of Health Sciences and TechnologyETH ZürichZürichSwitzerland

**Keywords:** motivation, exercises, aged, tablet, mobility, delivery of health care

## Abstract

**Background:**

Reaction time, coordination, and cognition performance typically diminish in older adults, which may lead to gait impairments, falls, and injuries. Regular strength–balance exercises are highly recommended to reduce this problem and to improve health, well-being, and independence in old age. However, many older people face a lack of motivation in addition to other strong barriers to exercise. We developed ActiveLifestyle, an information technology (IT)-based system for active and healthy aging aiming at improving balance and strength. ActiveLifestyle is a training app that runs on a tablet and assists, monitors, and motivates older people to follow personalized training plans autonomously at home.

**Objective:**

The objectives were to (1) investigate which IT-mediated motivation strategies increase adherence to physical exercise training plans in older people, (2) assess the impact of ActiveLifestyle on physical activity behavior change, and (3) demonstrate the effectiveness of the ActiveLifestyle training to improve gait speed.

**Methods:**

A total of 44 older adults followed personalized, 12-week strength and balance training plans. All participants performed the exercises autonomously at home. Questionnaires were used to assess the technological familiarity and stage of behavior change, as well as the effectiveness of the motivation instruments adopted by ActiveLifestyle. Adherence to the exercise plan was evaluated using performance data collected by the app and through information given by the participants during the study. Pretests and posttests were performed to evaluate gait speed of the participants before and after the study.

**Results:**

Participants were 75 years (SD 6), predominantly female (64%), held a trade or professional diploma (54%), and their past profession was in a sitting position (43%). Of the 44 participants who enrolled, 33 (75%) completed the study. The app proved to assist and motivate independently living and healthy older adults to autonomously perform strength–balance exercises (median 6 on a 7-point Likert scale). Social motivation strategies proved more effective than individual strategies to stimulate the participants to comply with the training plan, as well as to change their behavior permanently toward a more physically active lifestyle. The exercises were effective to improve preferred and fast gait speed.

**Conclusions:**

ActiveLifestyle assisted and motivated independently living and healthy older people to autonomously perform strength–balance exercises over 12 weeks and had low dropout rates. The social motivation strategies were more effective to stimulate the participants to comply with the training plan and remain on the intervention. The adoption of assistive technology devices for physical intervention tends to motivate and retain older people exercising for longer periods of time.

## Introduction

### Background

The primary goal of public health care is to increase the number of years of good health and maintain independence and quality of life as long as possible. Healthy aging is characterized by the avoidance of disease and disability, the maintenance of high physical and cognitive function, and sustained engagement in social and productive activities. These 3 components together define successful aging [1].

An important part of successful aging is maximization of physical performance. The ability to fully participate in productive and recreational activities of daily life may be affected when the capacity to easily perform common physical functions decreases [1]. Thus, health status is an important indicator of quality of life among older people [2,3]. It has been demonstrated that components of health-related fitness and functional performance or serious, chronic conditions and diseases that directly influence the components of fitness and performance are related to perceived health among middle-aged and older adults [3-5].

Regular physical activity or exercise substantially prevents the development and progression of most chronic degenerative diseases [6-8], is of benefit to frail and older persons, and is the only therapy found to simultaneously improve sarcopenia, physical function, cognitive performance, and mood in older adults [9]. For older people, a sedentary lifestyle also increases the risk of falls, whereas physically active older people have a reduced risk of falls with injuries [10-12]. An important marker for improvements in physical function that influences health and survival is gait speed [13]. In summary, to increase older adults’ quality of life and fitness, we need to encourage them to become or stay physically active [14-15] and increase their fitness through training.

The objective of this research is to run a phase II study [16] with a tablet app called ActiveLifestyle [17], an app for the autonomous strength-balance physical training for independently living older adults. We aimed to investigate (1) which information technology (IT)-mediated motivation strategies increased adherence to physical exercise training plans in older people, (2) whether these strategies could induce physical activity behavior change, and (3) the effectiveness of ActiveLifestyle training to improve gait speed.

### Related Work

Home environmental interventions to prevent functional decline seem to be effective [18] and are preferred by older people (ie, instead of leaving their houses to exercise) [19]. Interventions with integrated assistive technology devices have, in this context, the potential to further help in overcoming some of the barriers to start training [20] and maintaining physical independence for independently living older people [21]. Recently developed innovative ideas designed to alter clinical practice in sports were based on the development of tablet apps for prevention, for instance [22]. Tablet and smartphone software apps specifically designed for health purposes are, in general, enthusiastically adopted as a means of delivering self-managed health interventions [23-25]. However, such tablet-based interventions are often plagued by high attrition rates and varying levels of user adherence [24,25]. Furthermore, the effectiveness of tablet-based health intervention approaches has not yet been demonstrated in older people.

From a pilot study, we knew it is feasible to use assistive technology devices in an older population with the aim of encouraging performance of physical exercise [20]. The short-duration pilot did not focus on aspects of physical functioning, but indicated that the app could be improved by explicitly considering additional motivational strategies. It is well known that motivation strategies affect adherence to health interventions [26]; however, only a few solutions explore different motivation techniques to stimulate regular physical activity [26-28]. Most of these solutions have the drawback that they do not specifically focus on older people. Albaina et al [29] presented a user-friendly software interface running on a small touchscreen display to motivate older adults to walk. The authors used a graphical representation of a flower to motivate and assist seniors to monitor their daily amount of steps collected by pedometers through this simple metaphor of their performance. To the best of our knowledge, there is not another software app dedicated to strength-balance training plans for older people.

## Methods

### ActiveLifestyle

ActiveLifestyle is a software app for active aging, aimed at assisting, monitoring, and motivating older people during autonomous home-based physical workouts [20,30,31]. The software takes usability aspects into account, to ensure that older users can use it independently and it adopts a set of motivation strategies to stimulate users to exercise regularly. A video of the app is available on YouTube [32], and the app can be downloaded from the Apple App Store [17].

**Figure 1:**
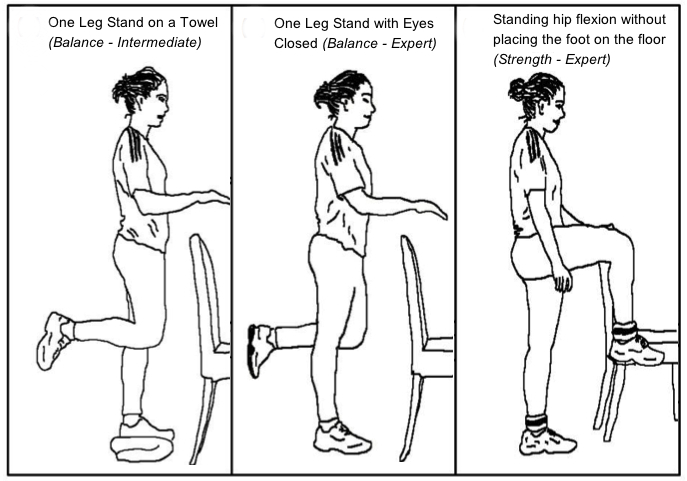
Exercise examples.

Three levels of strength-balance training plans are supported in the app: beginner, intermediate, and expert. In all levels, the balance training should be done 5 days per week. Sessions are composed of 3 exercises, in which the trainees repeatedly (1-3 times) hold a certain position for several seconds (15-30 sec). Each level has different exercises, allowing progression as the person advances through the levels (eg, at the intermediate level the older person must perform the exercises while standing on a towel; at the expert level the exercise must be performed with the eyes closed). Strength training has 3 levels and should be done twice a week; starting with 6 warm-up exercises, then 10 strength and 2 stretching exercises. A minimum number of sets (1-3) and repetitions (12-30) are available for each exercise. Some exercises require the use of weights (2-6 kg). The required effort of the exercises increases according to the level (eg, the beginner level does not require weights; the intermediate level requires ankle weights and performance in the sitting position; the expert level requires weights and exercises performed in standing position). The strength-balance training follows best practices recommendations and training principles (eg, it is progressive in nature) [33,34]. Figure 1 illustrates some exercises supported by ActiveLifestyle. All exercises are available on YouTube [35].

**Figure 2:**
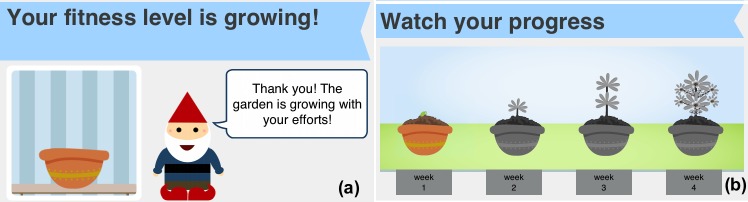
Metaphors within the app to motivate older people through conditioning, goal setting, and self-monitoring.

**Figure 3:**
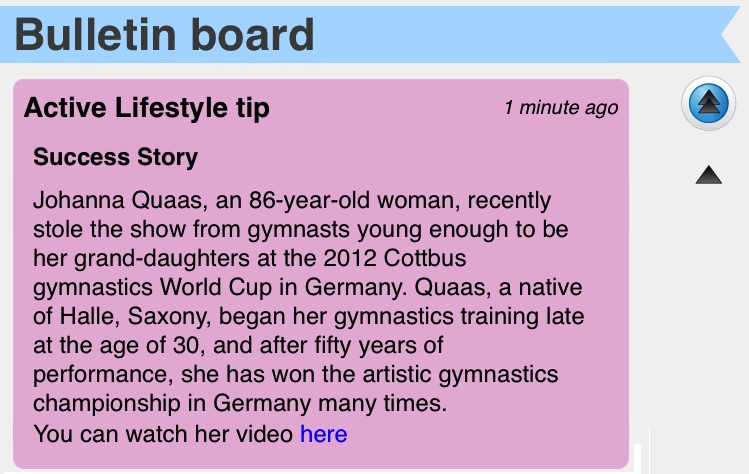
ActiveLifestyle tips to improve awareness about the benefits of being physically active.

In addition to the actual physical training, ActiveLifestyle features a set of individual and social motivation instruments. In general, individual motivation strategies aim to convince someone to do something because it is inherently enjoyable for this person, independently of any social pressure. ActiveLifestyle specifically supports:

1. Conditioning through positive and negative reinforcement by immediately offering a reward/praise after an expected behavior to encourage the behavior and increase the probability that it happens again, or reprimands undesired behavior to decrease the probability of a reoccurrence of that behavior. Metaphors for reinforcement include a flower that grows whenever a session is completed (ie, positive reinforcement) and a gnome who takes care of the flower. The gnome’s mood status varies according to the person’s daily compliance to the plan (ie, positive and negative reinforcement; if the person performs the exercise, the gnome is happy, otherwise he is sad) (see panel a in Figure 2).
2. Goal setting by establishing specific, measurable, achievable, and time-targeted goals. The goal is anticipated by visually conveying the achievable maximum growth of the flower (see panel b in Figure 2).
3. Self-monitoring by allowing people to monitor themselves and to modify their attitudes and behaviors. Coloring the flower growth stages reflects progress toward the goal (see panel b in Figure 2).
4. Awareness by presenting the benefits of being physically active through written content on a bulletin board and by showing inspiring stories (eg, link to newspapers, videos, or websites) (Figure 3).

**Figure 4:**
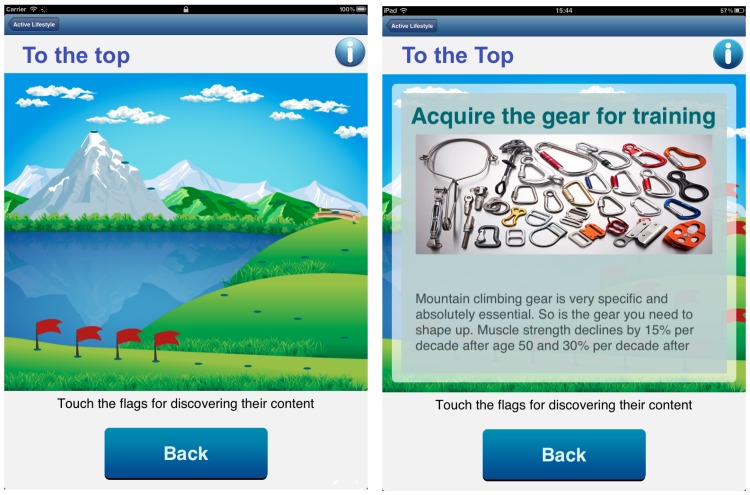
The To the Top collaboration game within the app.

Social motivation strategies are built on social psychology. An individual’s social network (other trainees) may act as source of motivation. ActiveLifestyle uses:

1. Comparison by allowing a person to compare similarities and differences between 2 or more parties. People tend to keep equality in their relationships. Whenever a person completes a workout session, an automatic message is posted on a bulletin board informing the training community (ie, other users following the same training plans) about the complete session. The message also carries the status of the individual’s flower.
2. External monitoring by allowing 1 party to monitor the performance of another party. ActiveLifestyle enables health care experts to access data on performance and compliance with the training plan. The older users have access to their own flower and to that of their training partners, enabling monitoring progress of peers.
3. Emotional support by encouraging exchange of written messages between trainees and experts to motivate and assist. ActiveLifestyle uses a bulletin board and an “inBox.” The first is a public channel where all members of the training community have access. The second is a bidirectional private channel for contact with professionals capable of giving advice and feedback on trainings.
4. Collaboration by offering a collaborative activity designed as a game, in which to progress in the game, a group of trainees must jointly be compliant with the training plan. The To the Top game is a trekking trail with 24 predefined points (2/week). The aim of the game is to climb a mountaintop, as a group of successful trainings ends. Compliance with the training plan is evaluated twice weekly on group level. A total of 65% or more members of a group have to perform the scheduled workout to be awarded a new flag on the trail (representing progress toward the mountaintop). Each flag uncovers a story with trivia about the Matterhorn and what is needed to conquer the mountain as a parable explaining the benefits associated with being physically active (Figure 4).

ActiveLifestyle comes in 2 versions. The individual version contains only the individual motivations strategies. The social version supports individual and social motivation strategies, and a virtual training plan community and communication features. In addition to the motivation strategies, ActiveLifestyle supports 6 main features accessible through its menu:

1. The What’s Next? option invites the users to start the performance of due workout sessions.
2. The weekly exercises option shows the scheduled strength–balance sessions organized per week.
3. The progress option shows the users’ progress thought the conditioning, goal setting, and self-monitoring strategies previously mentioned in both versions. The social version also supports the collaboration strategy through the To the Top game.
4. The bulletin board allows the users to receive written messages, which may include links for websites and YouTube videos. Three types of messages are supported: (1) workout session completed messages (in green) to inform the participant(s) about the conclusion of a scheduled session of exercises; (2) ActiveLifestyle tips messages (in pink) to support the awareness motivation strategy illustrated in Figure 3; and (3) public messages (in white) written by the training members. Only the social version supports the third type of message and has the ability to send messages to the entire training plan community.
5. The friends option lists the members of the training plan community (ie, older users and experts). Only the social version supports this feature.
6. The inBox option allows users to exchange private text messages with their list of friends.

All the previously mentioned features and motivation strategies can be inspected at the Life Participation Project website [31].

### Eligibility Criteria

Participants were older adults aged 65 years or older; living independently; able to walk independently with or without walking aids; able to follow instructions spoken in German, English, or Italian; and with no severe illness, cognitive impairment, progressive neurological disease, stroke, severe cardiac failure, or high blood pressure. Ethical approval for the study was obtained from the Eidgenössische Technische Hochschule (ETH) Ethics Committee (EK 2011-N-64).

### Setting

Participants were recruited by convenience sampling from 2 institutions for older people and 1 organization responsible for coordinating and providing at-home nursing care for seniors. The Senioren Begegnungszentrum Baumgärtlihof, a day center dedicated to delivering services and information related to the older population (Horgen, Switzerland), advised potential participants through its mailing list and by notes in the local newspaper (Multimedia Appendix 1). The Alterswohnungen Turm-Matt, a cooperative offering housing and daily living facilities to older people (Wollerau, Switzerland), informed and advised potential participants in person or by phone and distributed flyers to advertise the study. The Fachstelle für präventive Beratung Spitex-Zürich, a home-care nursing organization (Spitex-Zürich), promoted the study by sending letters and specifically inviting patients in need of better physical performance. Spitex-Zürich nurses selected potential participants based on the eligibility criteria.

### Intervention

**Figure 5:**
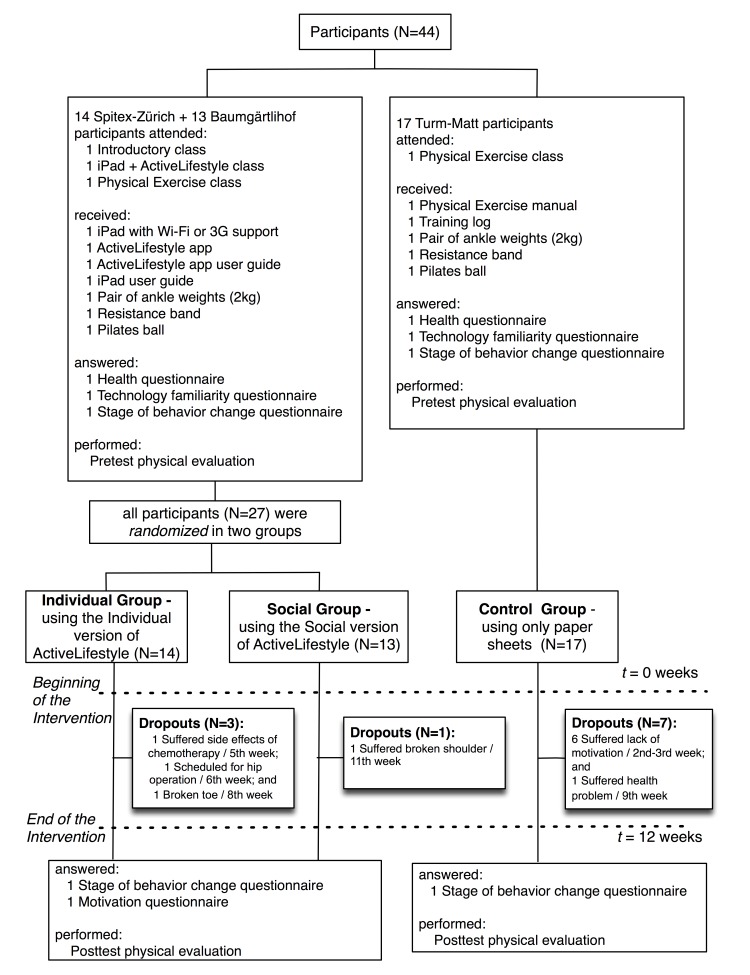
Flowchart of participants.

To investigate the effects of different motivation strategies, a pretest/posttest preclinical trial was performed. For convenience, the ActiveLifestyle groups were composed of (1) an individual group that followed training using the individual version of ActiveLifestyle; (2) a social group that followed training using the social version of the app, (3) a control group that followed exercises with printed information without additional motivation strategy. The individual and social groups were randomly composed of participants recruited from Baumgärtlihof and Spitex-Zürich, whereas the participants in the control group were recruited from Turm-Matt because of time and resource constraints (eg, lack of research team members, the control group was not randomized with the other participants). Figure 5 shows the recruitment process and the flow of participants through the study. Videos of some parts of the interventions can be watched on YouTube [35].

The development of our intervention follows a framework for the design and evaluation of complex interventions [36] and should at this stage be considered as a preclinical exploratory trial. For this reason, we did not use a pure randomized, controlled research design; therefore, we did not register this study as a clinical trial.

### Outcome Measures

#### Adherence and Attrition

Adherence was computed by ActiveLifestyle during the intervention and stored in a central database. The control group adherence was assessed with paper-based training logs. To calculate adherence, the total number of workout sessions for each participant was divided by 81, which was the total number of possible training sessions for the 12-week period (because of technical issues, the training was suspended for 3 days and the trainees were aware of the 81 training sessions in advance). The adherence of participants who dropped out was calculated by dividing the number of workout sessions attended up to the point of dropout from the study by 81 [37]. Values were compared between groups and with median rates in community-based fall prevention interventions [38]. For attrition, we measured the number of participants retained and lost at the final follow-up.

#### Gait Speed

The effect of the training on physical performance was assessed by measuring preferred and fast walking speed [39] with the GAITRite walkway, a valid and reliable tool for measuring gait in older people [40-42].

#### Motivation Instruments

The effectiveness of the motivation instruments built into the system was assessed based on the participants’ feedback, collected with a 7-point Likert scale self-reported questionnaire at the end of the intervention (Multimedia Appendixes 2 and 3), and on the performance (adherence, attrition, and gait speed) comparison among the 3 groups of participants.

#### Change of Behavior

The level of exercise adoption was evaluated according to the Transtheoretical Model (TTM) [43], which describes how people modify or acquire behavior. A self-reported TTM questionnaire (Multimedia Appendix 4) was applied before and after the training period. Participants were classified into 4 groups: contemplation (eg, thinking about physical behavior change), preparation (eg, already somewhat physically active), action (eg, doing enough physical activity), and maintenance (eg, making physical activity a habit).

#### Statistical Analyses

Analysis of variance (ANOVA) was used to test for differences in adherence to the training program between groups, as well as gait speed over time and between groups. Significant main effects were followed up by post hoc *t* tests with correction for multiple comparisons. Between-group differences in attrition were analyzed using a chi-square (χ^2^) test. Questionnaires on enjoyment, motivation, and change of behavior were analyzed using Kruskal–Wallis ANOVA and Wilcoxon signed rank tests (*W*). In all analyses, the level of significance was set at *P*≤.05. For effect size, we used η^2^ in all ANOVA analyses, Cohen’s *d* for all post hoc analyses, mean square contingency coefficient (φ) for chi-square tests, and Pearson *r* (*r*) for Kruskal–Wallis ANOVA and Wilcoxon signed rank tests. The *r* is calculated as *r*=Z/√N, in which Z is the standardized difference and N is the total number of samples. Suggested norms for interpreting η^2^ are 0.01=small, 0.06=moderate, and 0.14=large effect [44]. For small, moderate, and large effects, these norms are 0.2, 0.5, and 0.8, respectively, for Cohen’s *d* and 0.1, 0.3, and 0.5, respectively, for both φ and *r* [44]. All tests were conducted using SPSS Version 21.0 (IBM Corp, Armonk, NY, USA).

## Results

### Demographics

Detailed information about the participants’ demographics, based on the Health and Technology Familiarity self-reported questionnaires (Multimedia Appendixes 5 and 6), is summarized in Table 1.

### Adherence and Attrition

Table 2 presents the adherence to ActiveLifestyle strength–balance training plans. Adherence across training plans differed significantly between groups (*F*_2,41_=4.8, *P*=.01 η^2^=0.19). Post hoc *t* tests with Benjamini–Hochberg correction revealed a large and significant difference between the social group (mean 81.9%, SD 1.6%) and the control group (mean 48.1%, SD 41.5%; *t*_19.2_=3.1, *P*=.02 *d*=0.91). The difference between the individual group (mean 71.1%, SD 25.2%) and the control group was moderate to large (*t*_26.9_=1.9, *P*=.10, *d*=0.63). The difference between the individual and social groups was moderate yet nonsignificant (*t*_18.6_=1.4, *P*=.19, *d*=0.50).

**Figure 6:**
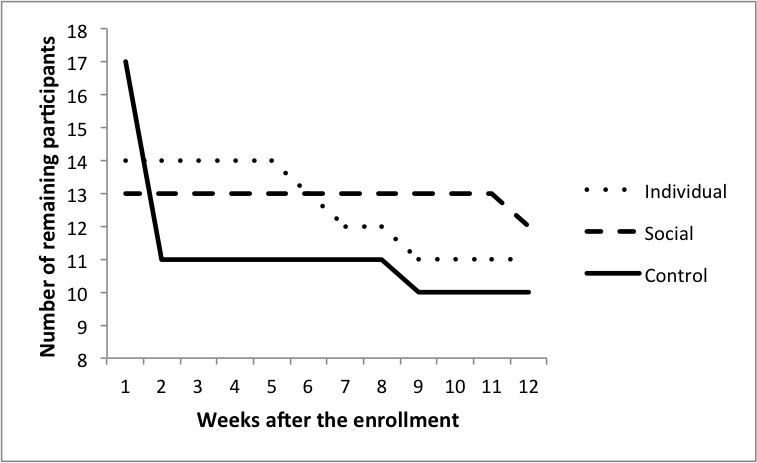
Graph of the number of remaining participants in each group per week.

Thirty-three older adults completed the 12 weeks of training, resulting in a 25% attrition rate in total, 21% in the individual group (3/14), 8% in the social group (1/13), and 41% in the control group (7/17). Figure 6 illustrates the number of remaining participants in each group per week after enrollment. More details about the dropout reasons are reported in Figure 5. A chi-square test revealed that attrition rate was higher in the control group (41.2%) than in the combined ActiveLifestyle groups (14.2%; χ^2^_1_=3.9, *P*=.05, φ=0.30).

**Table 1:** Participants’ demographics (N=44).

Characteristic		Individual (n=14)	Social (n=13)	Control (n=17)
Female gender, n (%)		10 (71)	8 (62)	10 (59)
Age (years), mean (SD)		74 (5)	75 (6)	76 (15)
Hold trades or professional diploma, n (%)		7 (50)	7 (54)	10 (59)
In a sitting position past profession, n (%)		7 (57)	6 (46)	6 (35)
**Health questions, n (%)**				
	Estimated good health	5 (36)	8 (61)	8 (47)
	Estimated average balance	7 (50)	5 (38)	9 (53)
	Feel pain but not every day	9 (64)	7 (54)	7 (41)
**Flexibility questions, n (%)**				
	Fell in the past 6 months^a^	2 (14)	5 (38)	4 (23)
	Walk at least twice a week	5 (36)	8 (61)	9 (53)
	Practiced some sport in the past	10 (71)	8 (61)	5 (29)
	Never practiced strength exercises	11 (79)	7 (54)	14 (82)
**Technology familiarity, n (%)**				
	Frequently use automated teller machines	7 (50)	9 (69)	7 (41)
	Frequently use cellphones	7 (50)	10 (77)	6 (35)
	Frequently use digital photography	8 (57)	4 (31)	4 (23)
	Don’t use Global Positioning System devices	7 (50)	8 (61)	6 (35)
	Don’t use automatic kiosks	9 (64)	6 (46)	12 (71)
	Don’t know what an e-book is	7 (50)	5 (38)	11 (65)
	Use a computer	12 (86)	10 (77)	8 (47)
	Between 1-5 hours per week	6 (43)	4 (31)	3 (18)
	Use the Internet	12 (86)	9 (69)	5 (29)
	Between 1-5 hours per week	7 (50)	6 (46)	2 (12)

^a^A fall was defined as unintentionally coming to the ground or some lower level, excluding the consequence of sustaining a violent blow, loss of consciousness, or sudden onset of paralysis, such as during a stroke or epileptic seizure [45].

**Table 2:** Adherence to ActiveLifestyle strength–balance training plans.

Training plan	Individual group			Social group			Control group		
	Visited	Planned	%	Visited	Planned	%	Visited	Planned	%
Balance training plan	547	812	67	549	754	73	451	986	46
Strength training plan	221	322	69	217	299	73	291	391	74
Across training plans	768	1134	68	766	1053	73	742	1377	54

### Gait Speed

Table 3 shows participants’ preferred and fast gait speed during the pretest and posttest evaluations. With respect to preferred gait speed, the 3 groups were similar. We used 2 mixed 2-way ANOVA’s (1 for preferred and 1 for fast gait) with within-subject factor pre–post (2 levels) and between-subject factor group (3 levels). For preferred gait speed, there was a significant difference between pretest and posttest (*F*_1,29_=29.5, *P*<.001, η^2^=0.50). Participants walked significantly faster in the posttest (1.276 m/s) than they did in the pretest (1.142 m/s). There was no significant main effect of group (*P*=.07) and no significant interaction effect (*P*=.65), suggesting that preferred gait speeds and their improvements were similar in all groups.

The results for fast gait speed were similar to those for preferred gait speed. Again, there was a large difference between pretest and posttest: Participants walked significantly faster in the posttest (1.72 m/s) than in the pretest (1.56 m/s; *F*_1,29_=20.1, *P*<.001, η^2^=0.41). The main effect of group was significant also (*F*_2,29_=5.3, *P*=.01 η^2^=0.27). Post hoc tests revealed that the individual group (1.89 m/s) was significantly faster than the control group (1.45 m/s; *t*_19_=3.94, *P*=.003, *d*=1.31), and faster than the social group (1.58 m/s; *t*_20_=2.05, *P*=.08, d=.89), though not statistically significant. The individual group, by chance, was the fastest from the beginning. Fast gait speed was not significantly different between the control group and the social group (*P*=.39).

### Motivation Instruments

Detailed information about the effectiveness of ActiveLifestyle’s motivation instruments and user-intention aspects are summarized in Table 4. The questionnaires used to collect the content of the table are available in Multimedia Appendixes 2 and 3.

Most participants affirmed that ActiveLifestyle facilitates the autonomous performance of balance–strength exercises. This was confirmed by a high intention to use the app again or to recommend it to friends or family members. The individual group was unanimous in the evaluation of these 2 user-intention aspects, whereas the social group presented high values but not with unanimity. In general, the participants of both groups did not feel motivated to perform physical exercises before the study.

All the participants thought it was fun to perform the strength and balance exercises. Few participants (<25%) felt frustrated, worried, or nervous during the study. More than half of the participants, 54% from the individual group and 67% from social group, will miss ActiveLifestyle.

The individual motivation strategies seemed to be more effective on the individual group level than on the social group level. Most of the individual group felt motivated by the goal-setting and self-monitoring strategies (91%), both represented by the progress bar metaphor (panel b in Figure 2), as well as for being aware of the benefits of being physically active–aware (82%). Conditioning through positive and negative reinforcement also motivated the participants. In all, 64% felt motivated when they saw the plant growing, whereas 55% felt motivated by the mood status of the gnome.

The most effective motivating strategies for the social group were conditioning through positive social inclusion and external monitoring (all 83%). After that, the social group felt motivated through the awareness of the benefits of physically activity (82%), emotional support (75%), the monitoring of their progress toward the plan (goal setting and self-monitoring) (67%), participation in the collaboration game (58%), positive and negative reinforcement (conditioning) (50%), and the comparison of their performance with other training participants on the bulletin board (42%).

Most participants in the individual group (64%) expressed that they would feel more motivated if they could use the social version of ActiveLifestyle, but the reverse was not true. Only a few participants in the social group expected to be less motivated using the individual version of the app (8%).

Mann–Whitney *U* tests comparing the Likert scores for all questions presented in Table 4 did not detect any significant differences between the groups.

### Change of Behavior

Table 5 shows the stage of behavior change of the participants at the beginning (t_0_=0 weeks) and at the end (t_1_=12 weeks) of the intervention.

Wilcoxon signed rank tests comparing pretest and posttest behavioral scores in each group revealed a trend—with a large effect size—in the social group (*W*=1.79, *P*=.07, *r*=0.52). Hence, the social group tended to change their behavior toward integration of ActiveLifestyle into their daily routine. No behavioral changes were detected in the control group (*P*=.28) or the individual group (*P*=.50). Although this suggests between group differences with respect to behavioral change, no such differences could be shown statistically; a Kruskal–Wallis ANOVA directly comparing change of behavior between the 3 groups was nonsignificant (*P*=.75).

**Table 3:** Participants’ gait speed during the pretests and posttests.

Condition		Pretest mean (SD)	Posttest mean (SD)
**Individual group**			
	Preferred speed (m/s)	1.26 (0.18)	1.42 (0.21)
	Fast speed (m/s)	1.80 (0.27)	1.98 (0.31)
**Social group**			
	Preferred speed (m/s)	1.10 (0.25)	1.24 (0.31)
	Fast speed (m/s)	1.50 (0.35)	1.66 (0.50)
**Control group**			
	Preferred speed (m/s)	1.07 (0.19)	1.17 (0.22)
	Fast speed (m/s)	1.39 (0.22)	1.51 (0.27)

**Table 4:** Outcome data expressed by the participants on a 7-point Likert scale (range 1-7; 1=completely disagree to 7=completely agree) at the end of the intervention period.

Evaluation statements		Individual (n=14)		Social (n=13)	
		Median (range)	% Agreed	Median (range)	% Agreed
**Statement**					
	ActiveLifestyle facilitates the performance of autonomous strength–balance exercises at home	7 (6-7)	100	7 (4-7)	92
**Use intention**					
	I would use the app again	6 (5-7)	100	6 (4-7)	83
	I would recommend the app to my friends and family	6 (6-7)	100	6 (3-7)	67
**Enjoyment**					
	It was fun to carry out the strength and balance exercises	6 (6-7)	100	6 (5-7)	100
	I felt frustrated during the study	2 (1-5)	9	2 (1-6)	8
	I felt worried during the study	2 (1-6)	18	2 (1-7)	25
	I felt nervous during the study	1 (1-6)	9	1 (1-4)	0
	I will miss the exercises and the ActiveLifestyle app	5 (2-7)	54	6 (3-7)	67
**Motivation**					
	I usually do not feel motivated to perform physical exercises, ActiveLifestyle helped me	6 (1-7)	54	6 (2-7)	83
**Individual motivation instruments**					
	I felt motivated when I saw my performance on the progress bar (goal setting and self-monitoring)	6 (4-7)	91	6 (1-7)	67
	I felt motivated by being aware about the benefits of being physically active (awareness)	6 (3-7)	82	6 (3-7)	82
	I felt motivated when I saw the plant growing due to my performance (conditioning)	6 (4-7)	64	6 (1-7)	83
	I felt motivated when I saw the emotional status of the gnome (conditioning)	5 (2-7)	55	4 (1-6)	50
	I would feel more motivated using the social version of ActiveLifestyle, in which I could interact with other training partners	5 (1-7)	64	—	—
**Social motivation instruments**					
	I felt motivated for being part of a training group and knowing that other people did the same exercises	—	—	6 (2-7)	83
	I felt motivated to perform the plan because I knew I was being monitored (external monitoring)	—	—	6 (2-7)	83
	I felt motivated for being emotionally supported by the other training partners and by the ActiveLifestyle experts (emotional support)	—	—	6 (2-7)	75
	I felt motivated with the collaboration activity to reach the top of the mountain (collaboration)	—	—	6 (3-7)	58
	I usually compared my flower with others on the bulletin board (comparison)	—	—	4 (1-6)	42
	I would feel more motivated using the individual version of ActiveLifestyle, which does not require interaction with other training partners	—	—	4 (1-6)	8

**Table 5:** Stage of behavior change of the participants according to the Transtheoretical Model (TTM).

Stage of behavior change		t_0_ = 0 weeks	t_1_ = 12 weeks
**Individual group**			
	Contemplation	3	0
	Preparation	1	4
	Action	1	1
	Maintenance	6	6
**Social group**			
	Contemplation	5	3
	Preparation	2	0
	Action	0	0
	Maintenance	5	9
**Control group**			
	Contemplation	5	3
	Preparation	1	2
	Action	0	0
	Maintenance	4	5

## Discussion

### Principal Findings

The aim of this study was to investigate (1) which IT-mediated motivation strategies increase adherence to physical exercise training plans in older people, (2) whether the ActiveLifestyle app induces physical activity behavior change, and (3) the effectiveness of the ActiveLifestyle training to improve gait speed. The main focus was to evaluate the ability to retain older people in the exercise program. Based on findings from a systematic review [39], we could expect a 10% attrition rate and 50% adherence rate for the individually targeted exercise training. Although the control group showed 41% attrition (primarily because of lack of exercise motivation), both tablet-based training groups showed far lower values, 21% and 8% for the individual and social ActiveLifestyle groups, respectively. These last 2 numbers also contain the effect of morbidities not related to the motivation to train (ie, unexpected health problems). Especially in the control group participants, the lack of motivation for continuous training was high. The degree of engagement with the intervention was more than 68% for the individual group and 73% for the social group, both using the ActiveLifestyle app, and 54% for the control group. Compared with median rates for attrition (10%) and adherence (50%) in fall prevention interventions in community settings, we achieved better or similar rates for the tablet-based training groups. From previous research [46], we know that the intention to undertake strength-balance training in older people is closely related to all elements of coping appraisal. Elements of coping appraisal include the belief that strength-balance training has multiple benefits, a positive social identity, and the feeling that family, friends, and doctors would approve of taking part in such training [46]. It can be hypothesized that ActiveLifestyle is effective in influencing attrition and adherence because it explicitly supports individual and social motivation instruments.

The reason to use a tablet solution is related to the numerous potential advantages attributed to such a tool (eg, tablets are relatively robust, and using fingers instead of a mouse or a touch pad make them much more intuitive and easy to use compared with smartphones, notebooks, and desktops). A tablet-based intervention, such as ActiveLifestyle, constitutes a powerful tool to provide feedback about performance and motivation to endure practice because of social inclusion. Interventions that use frequent, nonfrequent, or direct remote feedback are to be favored versus treatments without feedback, because the former seem to be more effective than the latter and they are equally effective as supervised exercise interventions [47]. The second most-mentioned barrier to physical exercise for subjectively insufficiently active older adults is lack of company. Direct remote contact seems to be a good alternative to supervised on-site exercising [47]. Such feedback can easily be adapted to the individual participant’s baseline motor performance and progressively augmented with task difficulty. ActiveLifestyle has been demonstrated to have the potential to engage people who otherwise would lack interest to participate in a physical exercise regimen. Especially in the older population, it is difficult to maintain high adherence to training programs [48]. The participants of the present study allocated to the tablet groups showed good compliance rates. The losses related to low exercise compliance (n=6) in the control training group were caused by a lack of motivation. The reasons for discontinuation of training in the tablet groups were not because of rejection of the app; they were because of health problems. In a future phase III trial, the follow-up period for the assessment of adherence and attrition should preferably be extended to 12 months to enable the comparability of this future study with reference values of previous physical interventions [39]. Although the result of a 12-week intervention, our findings are encouraging and indicate the effectiveness of a tablet-based training approach in older people. This encourages further exploration of this training approach in seniors.

Analyzing the participants’ answers to the motivation instruments of ActiveLifestyle, most of the individual participants (64%) would feel more motivated using the social version of the app, whereas the opposite is not true (8%) (both tablet groups were aware of the different versions of ActiveLifestyle). Regarding the physical activity habits, the training group using the social version of ActiveLifestyle was the only group showing a tendency to change behavior. At the end of the intervention, 50% of the social group participants changed their behavior according to the TTM. At the beginning, these participants were at the contemplation or preparation stages (thinking about or already being somewhat physically active), and they were classified as being on the maintenance stage (making physical activity a habit) by the end. However, a further longitudinal study with a larger sample, including evaluation after the end of the intervention, is required to be able to ascertain change of physical behavior.

Gait speed is a clinically relevant indicator of functional status associated with important geriatric health outcomes (ie, impact health care activities have on people) [49]. Slowing down has been recognized as an indicator of failing health and vulnerable old age [50]. Some researchers hypothesize that gait speed may act as a vital sign, giving indications of the health status of older people. Mortality, for example, is substantially reduced when gait speed is improved through interventions [51]. Large epidemiological studies reveal that a 0.1 m/s faster walking speed is related to a 12% decrease in mortality [13]. In this respect, it is encouraging that all older people in our training groups that adhered to their training plan, independently of their group allocation, showed an increase in both preferred and fast walking speed.

In addition to the high level of adherence caused by the social motivation instruments, the training community created by the study served to improve the connectedness of the participants, which may help people to garner social support for making physical changes in their daily lives [52]. Two women who did not know one another started to perform the exercises together to check if they were following the correct posture. Some participants contacted other training partners using the app or via email or phone when they faced problems. The same support was also requested from our team of experts, who frequently (especially at the beginning of the study) received phone calls because of technical problems or doubts about the exercises.

As learned in our previous study [20], some of the participants felt proud of being able to use new technology. One of our oldest participants (83 years) installed Skype to call his daughter living in Central America. He confessed that his daughter was very surprised. In the beginning, 1 woman was afraid of not being able to correctly operate the tablet because she had never used a computer before. After the study, she bought a tablet on her own to play with her grandchildren and installed Wi-Fi at home to be more connected with them. Another woman expressed a similar concern at the beginning of the training, but finished the study with a new tablet and a Gmail account: “I’m proud to be in possession of the iPad and to be able to write to my friends. The whole matter was a change for me.”

### Limitations

The study has some limitations. One of them is the rather small sample size. The study reveals first estimates for gait speed measures and stages of behavior change and warrants further research in larger populations. However, the purpose of preclinical exploratory trials is to provide preliminary evidence on the clinical efficacy of an intervention [16,36]. When evaluating the validity of a study, it is important to consider both the clinical and statistical significance of the findings [53]. Studies that claim clinical relevance may lack sufficient statistical significance to make meaningful statements or, conversely, may lack practicality despite showing a statistically significant difference in treatment options. Researchers and clinicians should not focus on small *P* values alone to decide whether a treatment is clinically useful; it is necessary to also consider the magnitude(s) of treatment differences and the power of the study [53]. Encouraging in this context is the observation that most of the between-groups comparisons for adherence show medium or medium-to-high magnitude(s) of treatment differences in favor of the tablet groups. The relationship between tablet-based physical training research and its effect on adherence and fitness in older individuals requires further exploration. Another limitation of this study is related to the research design used. The different recruitment methods and the lack of initial randomization and blinding may have introduced a selection bias that questions the validity of the adherence/motivation findings. Analogous studies with similar or frailer populations and the use of a true randomized controlled research design should be performed to substantiate or refute our findings.

The participants of this study can be classified as normal walkers with a preferred gait speed between 1.0 and 1.4 m/s. Future studies with community dwelling populations that exhibit mildly abnormal (0.6-1.0 m/s) or seriously abnormal gait speed (<0.6 m/s) [50] should be performed to investigate whether similar or even better results in physical performance variables can be obtained.

### Conclusion

The finding of this study supports the notion that it is advantageous to combine physical training with specifically targeted IT motivation instruments that offer the possibility to socialize in a group in clinical practice. The combination seems to have a positive influence on older adults’ training adherence in comparison to more traditional exercise. ActiveLifestyle proved to assist and motivate independently living and healthy older adults to autonomously perform strength–balance exercises. The social motivation strategies seemed to be more effective to stimulate the participants to comply with the training plan and remain on the intervention. The adoption of assistive technology devices for physical intervention tends to motivate and retain older people exercising for longer periods of time.

## Multimedia Appendix 1

Das iPad sagt Knie beugen: Zürichsee-Zeitung.

## Multimedia Appendix 2

Individual feasibility questionnaire.

## Multimedia Appendix 3

Social feasibility questionnaire.

## Multimedia Appendix 4

Transtheoretical-model questionnaire.

## Multimedia Appendix 5

Technology familiarity questionnaire.

## Multimedia Appendix 6

Health questionnaire.

## Abbreviations

ANOVA: analysis of variance
ETH: Eidgenössische Technische Hochschule
IT: information technology
TTM: Transtheoretical Model
