# Supplementary material for: Tablet-Based Strength-Balance Training to Motivate and Improve Adherence to Exercise in Independently Living Older People: A Phase II Preclinical Exploratory Trial
Source: J Med Internet Res. 2013 Aug 12;15(8):e159. doi: 10.2196/jmir.2579 (PMC3742406; doi:10.2196/jmir.2579)
Supplement: Supplementary file 1 [file jmir_v15i8e159_app1.pdf]

Das iPad sagt «Knie beugen»

HORGEN. Der Fitnesstrainer der Zukunft könnte portabel, orts- wie zeitungebunden sein. Die iPad-Applikation «Active Lifestyle App» ist von zwei jungen Wissenschafterinnen entwickelt worden. Senioren aus dem Bezirk testeten das Bewegungsprogramm.

GABY SCHNEIDER

Dass Senioren durchaus mit der modernsten Technik umgehen können, bewies eine aufgestellte Gruppe im AHV-Alter. Sie nahm am Montag an einem Apéro ihr Diplom für ein dreimonatiges iPad-angeleitetes Bewegungsprogramm entgegen. Dazu geladen hatte das Seniorenbegegnungszentrum Baumgärtlihof, welches das Projekt eines Kraft- und Gleichgewichtstrainings mittels iPad unterstützt hatte.

Balance und Kraft trainieren

«Stehen Sie auf einem Bein», verlangt die Fitness-App. Danach gibt der Senior sein Feedback ein, wie es ihm gefallen hat und wie oft er oder sie die Übungen durchgeführt hat. «Mir hat das Programm sehr gefallen», sagte Hans Rudolf Grunauer aus Wädenswil. «Bei gewissen Übungen kam ich richtig ins Schwitzen, besonders beim Krafttraining», berichtete der 72-Jährige. Ansprechend fand er auch, dass nach Absolvierung der Übungen ermutigende Belobigungen eintrafen. Sein «Fitnessbäumchen» wurde von Monat zu Monat grösser.

Die Übungen steigerten sich. «Für das Krafttraining brauchte ich im dritten Monat eine Dreiviertelstunde», sagt Grunauer. Er will mit diesem virtuellen Turnunterricht fortfahren. «Mir gefiel, dass ich nicht an eine Zeit gebunden war, wenn ich trainieren wollte», sagte Rosmarie Egolf aus Oberrieden. Das Ehepaar Marlène und Kurt Brüschweiler trainierte mit dem iPad zu Hause und ging dazu noch ins Fitnesscenter. «Ich war am Anfang unterfordert, erst am Schluss hat es mir was gebracht», äusserte sich Kurt Brüschweiler.

Zehn Personen aus Horgen und Umgebung nahmen teil, die fünfmal pro Woche den Lektionen aus dem iPad folgten. 66-jährig ist der jüngste und 82 die älteste Teilnehmerin. Begonnen hatte das Training im August. Zuvor waren die Teilnehmenden ganz real in die Bewegungsübungen eingeführt worden. Das iPad war ihnen gratis zur Verfügung gestellt worden, ebenso die Turngeräte wie Thera-Band, Fussgewichte und Pilates-Ball.

Kontrollgruppe in Wollerau

Muskeln kräftigen konnten die Senioren beispielsweise, indem sie sich ein 2-Kilo-Gewicht an die Beine schnallten. Das iPad zeigte den korrekten Bewegungsablauf. «Am Anfang schaute ich noch viel auf das Gerät, nachher konnte ich die Übung auswendig», berichtete Hans Rudolf Grunauer. Damit die Zuhause-Sportler nicht «vereinsamen», wurde per App der Austausch der Turner untereinander gefördert. Die Teilnehmer konnten sich jederzeit Nachrichten schicken. Mit einem einfachen Fingerstupf war man schnell beim Kollegen oder der Kollegin.

«Dieses Programm eignet sich gut für Leute, die nicht gern ins Fitnesscenter gehen», sagte Claudia Nüesch, Assistentin Zentrumsleitung im Baumgärtlihof. «Mit zwei Gruppen haben wir das Programm getestet», erläuterte Eva van het Reve, Doktorandin an der ETH, «mit einer in Horgen und einer zweiten Gruppe in Zürich.» Die Bewegungswissenschafterin hat die Applikation zusammen mit Patricia Silveira von der Universität Trento (Italien) entwickelt. Parallel zu den Gruppen in Horgen und Zürich trainierte eine Kontrollgruppe in Wollerau, allerdings nicht angeleitet vom iPad, sondern via gedrucktem Trainingsprogramm.

Die Ergebnisse werden nun wissenschaftlich ausgewertet. «Wir wollen feststellen, ob sich Kraft und Balance verbessert haben», erklärt Eva van het Reve. Beispielsweise müssen die Teilnehmer auf einem mit 200 000 Sensoren ausgestatteten Laufband gehen. Dabei werden ihre mobilen Fähigkeiten gemessen. Für die diplomierten iPad-Turner steht bereits jetzt fest, dass sie sich fitter als vor drei Monaten fühlen.

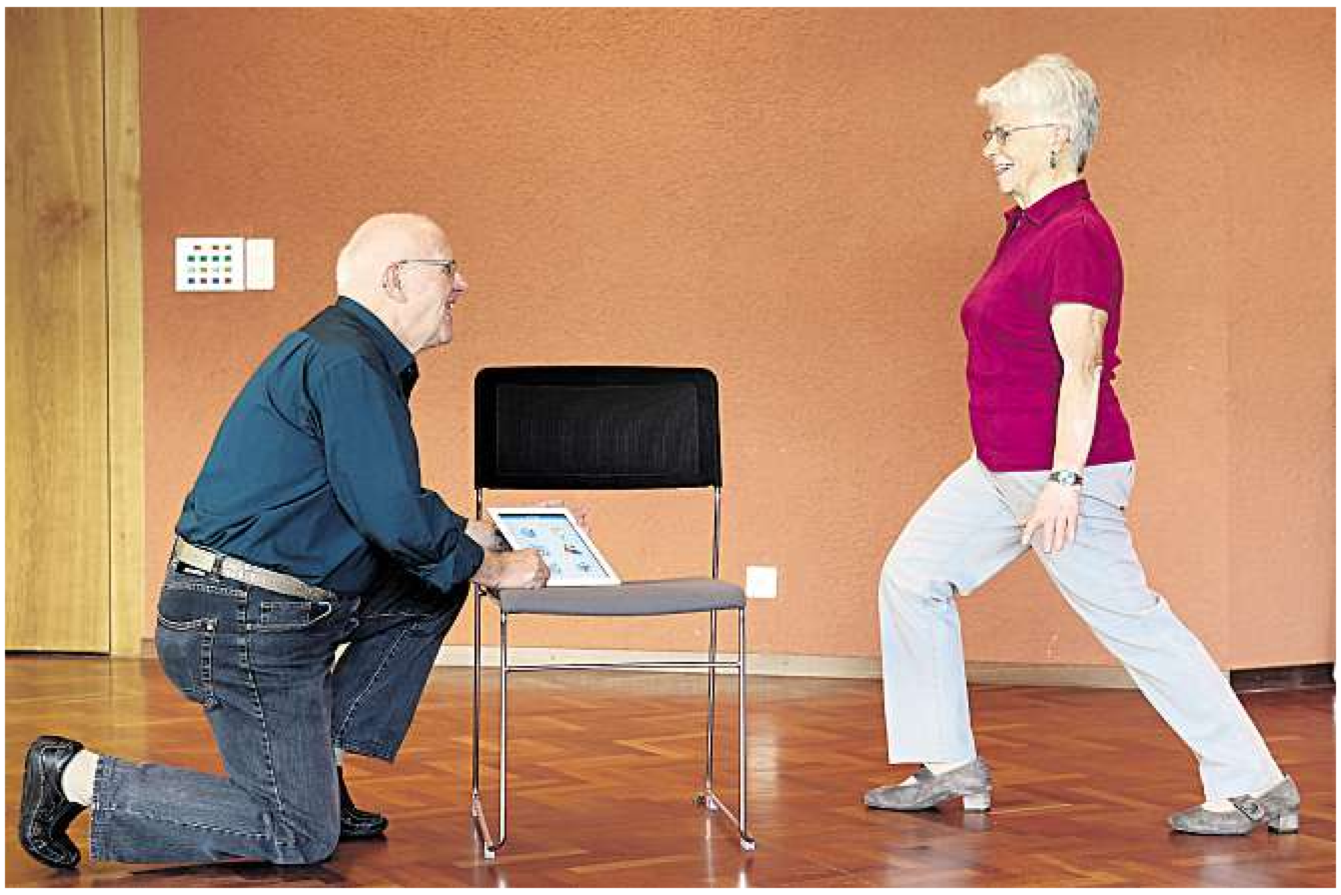

Das iPad liegt auf dem Stuhl. Kurt und Marlène Brüschweiler folgen den Anweisungen. Bild: Silvia Luckner

Palmisackerstrasse gesperrt

SCHÖNENBERG. Infolge Sanierungsarbeiten bleibt die Palmisackerstrasse in Schönenberg heute Mittwoch für den Verkehr vollständig gesperrt. Dies teilt das Werkhof-Team der Gemeinde Schönenberg mit. Ist das Wetter schlecht, verschieben sich die Arbeiten auf den nächsten Tag. Wer Fragen dazu hat, wendet sich an den Strassenmeister Max Schärer, Telefon 079 447 73 85. *(zsz)*

VERANSTALTUNGEN

Personalverordnung ändern

RICHTERSWIL. Heute Mittwoch lädt der Gemeinderat Richterswil zu einer ausserordentlichen Gemeindeversammlung ein. Behandelt wird lediglich ein Geschäft: die Änderung der Personalverordnung. Die Gemeindeversammlung soll dem Gemeinderat die Kompetenz delegieren, die Personalvorsorge für die Mitarbeitenden der Gemeinde selbst zu wählen. Sagen die Stimmberechtigten dazu Ja, könnte die Gemeinde Richterswil aus der Beamtenversicherungskasse (BVK) austreten und seine Angestellten von der Swiss Life versichern lassen. *(zsz)*

Mittwoch, 7. November, 20 Uhr, reformierte Kirche Richterswil.

Hip-Hop-Nacht

HORGEN. Am kommenden Freitag regiert König Hip-Hop das See la vie. Der Horgner Didi und sein Zürcher Kumpel Ikara präsentieren ihr erstes Album «Karma». Die beiden arbeiten seit 2008 zusammen, das erste gemeinsame Projekt erschien unter dem Namen «Dä Batze muess stimme». Erst im Frühling traten Ikara und Didi als Anheizer für Skor und Steezo in Horgen auf, jetzt zeigen die Rapper, dass sie sich nicht zu verstecken brauchen. Vor dem Hauptact stehen 8810 Recordz auf der Bühne. Die vier Rapper Daki, Vali-G, Ché und Kevin51 machen seit etwa einem Jahr zusammen Musik und haben, wie der Name schon verrät, ein Heimspiel. *(e)*

Freitag, 9. November, 20.30 Uhr, See la vie, Alte Landstrasse 26, Horgen.

LESERBRIEFE

Breite Diskussion gewünscht

Zur Teilrevision der Gemeindeordnung Horgens

In Horgen nimmt die Aufsicht über Amtsführung des Gemeinderats, Budget und Rechnung die Gemeindeversammlung wahr. Sie wird dabei von der Geschäftsprüfungskommission beraten. Die Bedeutung der Gemeindeversammlung ist in diesem System sehr hoch. Sie tagt zwar nur zwei bis dreimal jährlich, aber diese neun bis zwölf Stunden müssen genügen. Die Frage, ob diese Form bei einer Gemeindegrösse von bald 20 000 Einwohnern an Grenzen stosse, steht seit einiger Zeit im Raum. Mit der Teilrevision der Gemeindeordnung, die der Gemeinderat bis zum 15. November in die Vernehmlassung gegeben hat, verschärft sich diese Frage. Der Vorschlag sieht einen Machtzuwachs des Gemeinderates auf Kosten des Stimmvolkes vor:

Das Volk wählt nur noch sechs statt neun Personen in den Gemeinderat. Dazu kommt das Schulpräsidium. Auch für die Schulpflege sollen zwei Personen weniger gewählt werden. Anstelle der vom Volk gewählten Sozialbehörde will der Gemeinderat selber eine beratende Sozialkommission einsetzen. Die Aufgaben im Altersbereich sollen mit Vermögenswerten der Gemeinde in eine Stiftung ausgelagert werden. Der Stiftungsrat wird vom Gemeinderat gewählt.

Mir scheint, dass eine solche Konzentration der Entscheidungsmacht ohne Stärkung der Aufsicht fahrlässig ist. Die demokratische Bodenhaftung geht verloren. Ich liebe die Gemeindeversammlung und nehme regelmässig daran teil. Mit den geplanten Neuerungen wären meine Interessen gegenüber dem Gemeinderat allerdings besser aufgehoben, wenn ich sie einem gewählten Gemeindeparlament übertragen könnte. Dieses würde die Gemeindegeschäfte auch dann begleiten, wenn Fussballmeisterschaften stattfinden oder ein Schneewetter das Stimmvolk an der Teilnahme der Gemeindeversammlung hindert. Mit immer weniger gewählten Behördenmitgliedern verkommt die Gemeindeversammlung zum Alibi-Ritual für einen kleinen Gemeinderat, der unser Gemeinwesen weitgehend autokratisch managt.

Ich wünschte mir darüber eigentlich eine breite Diskussion mit Beteiligung aller politischen Parteien und aller Bürgerinnen und Bürger in Horgen.

Johannes Bardill, Horgen

Junge Kandidaten für Exekutive

Zu den Adliswiler Stadtrats-Ersatzwahlen vom 25. November

Als junge Wählerin möchte ich bei den Adliswiler Stadtrats-Ersatzwahlen vom 25. November junge, dynamische und zukunftsorientierte Personen in unserer Stadtregierung sehen, die sich für die Wünsche und Bedürfnisse von jungen wie auch von älteren Menschen einsetzen und sich so für einen generationenübergreifenden Dialog eignen. Mit seinen 31 Jahren ist Renato Günthardt noch jung, was für mich aber keinen Nachteil darstellt. In der Stadt Schaffhausen wurde vor wenigen Wochen ein 31-Jähriger in den Stadtrat gewählt, und in einer St. Galler Gemeinde wurde ein Kandidat gleichen Alters sogar Gemeindepräsident. Trotz seines noch jungen Alters hat Renato Günthardt sowohl Führungserfahrung in einer öffentlichen Verwaltung, die er während sechs Jahren als Leiter des Gemeindeammann- und Betreibungsamtes von Affoltern am Albis gesammelt hat, als auch politische Erfahrung durch seine langjährige Mitgliedschaft im Gemeinderat. Solche jungen Politiker verdienen eine Chance.

Manuela Honegger, Adliswil

Viele besuchen die Oberriedner Badi

Zum Leserbrief «Sehr zufrieden mit Bademeister», Ausgabe vom 9. Oktober

Die Interpellation der GLP Wädenswil zur Badi Rietliau begrüsse ich sehr. Die Meinung, es ist doch gar nicht so schlimm, wenn es spitzige Steine und scharfe Muscheln im Nichtschwimmerbereich hat, halte ich für gelinde gesagt ziemlich zynisch. Tatsache ist, meine Tochter, sie ist sechs Jahre alt, hat sich schon mehrfach die Füsse verletzt. Wir haben das Problem so gelöst, dass wir nach Oberrieden in die Badi fahren. Da stimmt das Restaurant und der Nichtschwimmerbereich.

Fahren Sie einmal an einem schönen Sommertag nach Oberrieden und zählen Sie die Wädenswiler Familien, die dort in der Badi sind. Sie werden staunen. Schade eigentlich, hat doch unsere Badi das Potenzial, zu den Besten am Zürichsee zu gehören.

Rolf Nessler, Wädenswil

Grüne Horgen fordern ein Parlament

HORGEN. Die Grünen hätten sich eine breite öffentliche Debatte zur Teilrevision der Gemeindeordnung gewünscht. Dies schreiben sie in einer Mitteilung. Der Gemeinderat habe die Chance verpasst, die Einführung eines Parlaments zur Diskussion zu stellen.

Nach Ansicht der Grünen ist eine Gemeindeversammlung für den Bezirkshauptort mit bald 20 000 Einwohnern nicht mehr zeitgemäss. Es brauche eine Legislative, die sich vertieft mit anstehenden Geschäften beschäftigen und die Exekutive entsprechend kontrollieren kann. Parlamentsmitglieder spezialisieren sich auf gewisse Themen, mit denen sie sich über längere Zeit hinweg befassen.

In ihrer Vernehmlassungsantwort äussern sich die Grünen kritisch gegenüber einer Verkleinerung des Gemeinderats. Ein solcher Schritt hätte eine grössere zeitliche Belastung der einzelnen Exekutivmitglieder zur Folge. Für Horgner, die mitten im Berufsleben stehen, käme ein solches Amt aus zeitlichen und finanziellen Gründen kaum mehr in Frage. Neben der sozioökonomischen litte auch die parteipolitische Repräsentanz. Vertreterinnen und Vertreter kleinerer Parteien hätten viel schlechtere Chancen, gewählt zu werden.

Ferner lehnen die Grünen die Auslagerung der gemeindeeigenen Altersinstitutionen in eine Stiftung ab. Dadurch würde die demokratische Kontrolle über diesen Bereich eingeschränkt. Mit dem grösseren unternehmerischen Spielraum erhielten Wirtschaftlichkeitsüberlegungen mehr Gewicht. Es besteht die Gefahr, dass Leistungen der Angestellten unter Druck geraten. Alle übrigen Änderungsvorschläge unterstützen die Grünen: die Integration des Schulpräsidiums in den Gemeinderat, die Verkleinerung der Mitgliederzahl der Schulpflege, die Umwandlung der Sozialbehörde in eine vom Gemeinderat bestellte, beratende Sozialkommission sowie die Verselbständigung der Pensionskasse. *(e)*

CVP will Strasse sanieren

HORGEN. An ihrer kürzlich abgehaltenen Parteiversammlung hat sich die CVP Horgen mit den Urnengeschäften vom 25. November befasst. Die Sanierung der Plattenstrasse ist aus der Sicht der CVP notwendig und die Kosten vertretbar. Deshalb wird die Vorlage zur Annahme empfohlen.

Grundsätzlich wird das Projekt «Step by Step» als sehr gutes Projekt wahrgenommen. Jedoch äussert die CVP Bedenken bezüglich der Finanzierung für die Schüler aus anderen Gemeinden, welche noch nicht geklärt ist. Auch die Freiwilligkeit der Teilnahme bei «Step by Step» gibt Anlass zur Diskussion, denn es müssten auch Lösungen für diejenigen Schüler gefunden werden, die am Projekt kein Interesse zeigen.

Die CVP Horgen erteilt zudem den «Mieterschutzvorlagen» eine Abfuhr und unterstützt den vom Kantonsrat ausgearbeiteten Gegenvorschlag zur «Prima-Initiative». Die CVP unterstützt zudem die Änderung des Tierseuchen-Gesetzes. *(e)*
