# Supplementary material for: Tablet-Based Strength-Balance Training to Motivate and Improve Adherence to Exercise in Independently Living Older People: A Phase II Preclinical Exploratory Trial
Source: J Med Internet Res. 2013 Aug 12;15(8):e159. doi: 10.2196/jmir.2579 (PMC3742406; doi:10.2196/jmir.2579)
Supplement: Supplementary file 2 [file jmir_v15i8e159_app2.pdf]

Eidgenössische Technische Hochschule Zürich
Swiss Federal Institute of Technology Zurich

IBWS Institut für Bewegungswissenschaften und Sport

HIT J 32, Wolfgang-Pauli-Strasse 27, CH-8093 Züric
Eva van het Reve

Questionnaires to evaluate the Active Lifestyle Application

1. Details to be filled by the interviewer

Name of the interviewed:
Date:

2. Please answer the questions below taking in to account your two weeks training plan using the Active Lifestyle Application

2.1. Perceived Usefulness ("the degree to which a person believes that using a particular system would enhance his or her performance")

PU1 ActiveLifestyle facilitates me to perform the strength-balance exercises autonomously at home.

O Completely agree
O Agree
O Partially agree
O Neutral
O Partially disagree
O Disagree
O Strongly disagree

2.2. Use intention ("a course of action that one intends to follow")

INT1 I would use the application again.

O Completely agree
O Agree
O Partially agree
O Neutral
O Partially disagree
O Disagree
O Strongly disagree

INT2 I would recommend the application to my friends and family

O Completely agree
O Agree
O Partially agree
O Neutral

IBWS Institut für Bewegungswissenschaften und Sport
HIT J 32, Wolfgang-Pauli-Strasse 27, CH-8093 Züric
Eva van het Reve

	O Partially disagree<br>O Disagree<br>O Strongly disagree

2.3. Motivation ("providing with a reason to act in a certain way")

M1 I usually do not feel motivated to perform physical exercises, ActiveLifestyle helped me.	O Completely agree<br>O Agree<br>O Partially agree<br>O Neutral<br>O Partially disagree<br>O Disagree<br>O Strongly disagree
M2 I like the three metaphors (flower, tree, and garden)	O Completely agree<br>O Agree<br>O Partially agree<br>O Neutral<br>O Partially disagree<br>O Disagree<br>O Strongly disagree
M3 I felt motivated when I saw the plant growing due to my performance.	O Completely agree<br>O Agree<br>O Partially agree<br>O Neutral<br>O Partially disagree<br>O Disagree<br>O Strongly disagree
M4 I felt motivated when I saw the emotional status of the gnome.	O Completely agree<br>O Agree<br>O Partially agree<br>O Neutral<br>O Partially disagree<br>O Disagree<br>O Strongly disagree
M5 I felt motivated when I saw my progress on the bar.	O Completely agree<br>O Agree<br>O Partially agree<br>O Neutral

	O Partially disagree<br>O Disagree<br>O Strongly disagree
M6 I felt motivated for being aware about the benefits of being physically active informed by the ActiveLifestyle tips on the *Bulletin board*.	O Completely agree<br>O Agree<br>O Partially agree<br>O Neutral<br>O Partially disagree<br>O Disagree<br>O Strongly disagree
M7 I would feel more motivated using the Social version of ActiveLifestyle, in which I could interact with other training partners.	O Completely agree<br>O Agree<br>O Partially agree<br>O Neutral<br>O Partially disagree<br>O Disagree<br>O Strongly disagree

Please, order the list of motivation strategies below using a numeric scale. For instance, 1 for the most motivating strategy, and 5 for the least motivating strategy.

__

	the growing garden metaphor
	the emotional status of the gnome
	the progress on the bar
	the tips about the benefits of being active
	the fact of being monitored

2.4. Enjoyment

E1 It was fun to carry out the strength and balance exercises.	O Completely agree<br>O Agree<br>O Partially agree<br>O Neutral<br>O Partially disagree

O Disagree
O Strongly disagree

E2 I felt worried during the study.

O Completely agree
O Agree
O Partially agree
O Neutral
O Partially disagree
O Disagree
O Strongly disagree

E3 I felt nervous during the study.

O Completely agree
O Agree
O Partially agree
O Neutral
O Partially disagree
O Disagree
O Strongly disagree

E4 I felt frustrated during the study.

O Completely agree
O Agree
O Partially agree
O Neutral
O Partially disagree
O Disagree
O Strongly disagree

E5 I will miss the exercises and the ActiveLifestyle app

O Completely agree
O Agree
O Partially agree
O Neutral
O Partially disagree
O Disagree
O Strongly disagree
