# Supplementary material for: Tablet-Based Strength-Balance Training to Motivate and Improve Adherence to Exercise in Independently Living Older People: A Phase II Preclinical Exploratory Trial
Source: J Med Internet Res. 2013 Aug 12;15(8):e159. doi: 10.2196/jmir.2579 (PMC3742406; doi:10.2196/jmir.2579)
Supplement: Supplementary file 3 [file jmir_v15i8e159_app3.pdf]

Eidgenössische Technische Hochschule Zürich
Swiss Federal Institute of Technology Zurich

IBWS Institut für Bewegungswissenschaften und Sport

HIT J 32, Wolfgang-Pauli-Strasse 27, CH-8093 Züric
Eva van het Reve

Questionnaires to evaluate the Active Lifestyle Application

1. Details to be filled by the interviewer

Name of the interviewed:
Date:

2. Please answer the questions below taking in to account your two weeks training plan using the Active Lifestyle Application

2.1. Perceived Usefulness ("the degree to which a person believes that using a particular system would enhance his or her performance")

PU1 ActiveLifestyle facilitates the performance of the strength-balance exercises autonomously at home.

O Completely agree
O Agree
O Partially agree
O Neutral
O Partially disagree
O Disagree
O Strongly disagree

2.2. Use intention ("a course of action that one intends to follow")

INT1 I would use the application again.

O Completely agree
O Agree
O Partially agree
O Neutral
O Partially disagree
O Disagree
O Strongly disagree

INT2 I would recommend the application to my friends and family.

O Completely agree
O Agree
O Partially agree
O Neutral
O Partially disagree

O Disagree
O Strongly disagree

2.3. Motivation ("providing with a reason to act in a certain way")

M1 I usually do not feel motivated to perform physical exercises, ActiveLifestyle helped me.	O Completely agree<br>O Agree<br>O Partially agree<br>O Neutral<br>O Partially disagree<br>O Disagree<br>O Strongly disagree
M2 It was fun to carry out the strength and balance exercises.	O Completely agree<br>O Agree<br>O Partially agree<br>O Neutral<br>O Partially disagree<br>O Disagree<br>O Strongly disagree
M3 I like the three metaphors (flower, tree, and garden).	O Completely agree<br>O Agree<br>O Partially agree<br>O Neutral<br>O Partially disagree<br>O Disagree<br>O Strongly disagree
M4 I felt motivated when I saw the plant growing due to my performance.	O Completely agree<br>O Agree<br>O Partially agree<br>O Neutral<br>O Partially disagree<br>O Disagree<br>O Strongly disagree
M5 I felt motivated when I saw the emotional status of the gnome.	O Completely agree<br>O Agree<br>O Partially agree<br>O Neutral<br>O Partially disagree

	O Disagree<br>O Strongly disagree
M6 I felt motivated when I saw my progress on the bar.	O Completely agree<br>O Agree<br>O Partially agree<br>O Neutral<br>O Partially disagree<br>O Disagree<br>O Strongly disagree
M7 I felt motivated for being aware about the benefits of being physically active informed by the ActiveLifestyle tips on the Bulletin board and by the content of the flags on the To-the-top Game.	O Completely agree<br>O Agree<br>O Partially agree<br>O Neutral<br>O Partially disagree<br>O Disagree<br>O Strongly disagree
M8 It felt motivated for being part of a training group and to know that other people did the same exercises.	O Completely agree<br>O Agree<br>O Partially agree<br>O Neutral<br>O Partially disagree<br>O Disagree<br>O Strongly disagree
M9 I usually compare my flower with the other on the Bulletin board.	O Completely agree<br>O Agree<br>O Partially agree<br>O Neutral<br>O Partially disagree<br>O Disagree<br>O Strongly disagree
M10 I felt motivated to perform the plan because I knew that I was being monitored.	O Completely agree<br>O Agree<br>O Partially agree<br>O Neutral

Eidgenössische Technische Hochschule Zürich
Swiss Federal Institute of Technology Zurich

IBWS Institut für Bewegungswissenschaften und Sport

HIT J 32, Wolfgang-Pauli-Strasse 27, CH-8093 Züric
Eva van het Reve

O Partially disagree
O Disagree
O Strongly disagree

M11 I felt motivated with the collaboration activity to reach the top of the mountain.

O Completely agree
O Agree
O Partially agree
O Neutral
O Partially disagree
O Disagree

M12 I felt motivated for being emotionally supported by the other training plan partners and by the ActiveLifestyle experts.

O Completely agree
O Agree
O Partially agree
O Neutral
O Partially disagree
O Disagree
O Strongly disagree

M13 I would feel more motivated using the Individual version of ActiveLifestyle app, which does not require the interaction with other training partners.

O Completely agree
O Agree
O Partially agree
O Neutral
O Partially disagree
O Disagree
O Strongly disagree

Please, order the list of motivation strategies below using a numeric scale. For instance, 1 for the most motivating strategy, and 9 for the least motivating strategy.

__

	the growing garden metaphor
	the emotional status of the gnome
	the progress on the bar
	the tips about the benefits of being active
	the fact of being monitored
	the comparison of gardens on the bulletin board
	the mountain game
	the fact of being part of a training group
	the fact of being emotionally supported by the Active Lifestyle experts and my training partners.

IBWS Institut für Bewegungswissenschaften
und Sport
HIT J 32, Wolfgang-Pauli-Strasse 27, CH-8093
Züric
Eva van het Reve

2.4. Enjoyment

E1 It was fun to carry out the strength and balance exercises.	O Completely agree<br>O Agree<br>O Partially agree<br>O Neutral<br>O Partially disagree<br>O Disagree<br>O Strongly disagree
E2 I felt frustrate during the study.	O Completely agree<br>O Agree<br>O Partially agree<br>O Neutral<br>O Partially disagree<br>O Disagree<br>O Strongly disagree
E3 I felt worried during the study.	O Completely agree<br>O Agree<br>O Partially agree<br>O Neutral<br>O Partially disagree<br>O Disagree<br>O Strongly disagree
E4 I felt nervous during the study.	O Completely agree<br>O Agree<br>O Partially agree<br>O Neutral<br>O Partially disagree<br>O Disagree<br>O Strongly disagree
E5 I will miss the exercises and ActiveLifestyle.	O Completely agree<br>O Agree<br>O Partially agree<br>O Neutral<br>O Partially disagree

Eidgenössische Technische Hochschule Zürich
Swiss Federal Institute of Technology Zurich

IBWS Institut für Bewegungswissenschaften
und Sport

HIT J 32, Wolfgang-Pauli-Strasse 27, CH-8093
Züric
Eva van het Reve

O Disagree
O Strongly disagree
