# Supplementary material for: Tablet-Based Strength-Balance Training to Motivate and Improve Adherence to Exercise in Independently Living Older People: A Phase II Preclinical Exploratory Trial
Source: J Med Internet Res. 2013 Aug 12;15(8):e159. doi: 10.2196/jmir.2579 (PMC3742406; doi:10.2196/jmir.2579)
Supplement: Supplementary file 4 [file jmir_v15i8e159_app4.pdf]

Eidgenössische Technische Hochschule Zürich
Swiss Federal Institute of Technology Zurich

IBWS Institut für Bewegungswissenschaften und Sport
HIT J 32, Wolfgang-Pauli-Strasse 27, CH-8093 Züric

Eva van het Reve

Study. - Code:

Questionnaire

For each of the following questions, please circle **Yes** or **No**. Be sure to follow the instructions carefully.

Physical activity or exercise includes activities such as walking briskly, jogging, bicycling, swimming, or any other activity in which the exertion is at least as intense as these activities.

1. I am currently physically active. ☐ yes ☐ no
2. I intend to become more physically active in the next 3 months. ☐ yes ☐ no

For activity to be regular, it must add up to a total of 30 minutes or more per day and be done at least five days per week. For example, you could take one 30-minute walk/strength exercises or take three 10-minute walks for a total of 30 minutes.

3. I currently engage in regular physical activity. ☐ yes ☐ no
4. I have been regularly physically active for the past 3 months. ☐ yes ☐ no
