# Supplementary material for: Tablet-Based Strength-Balance Training to Motivate and Improve Adherence to Exercise in Independently Living Older People: A Phase II Preclinical Exploratory Trial
Source: J Med Internet Res. 2013 Aug 12;15(8):e159. doi: 10.2196/jmir.2579 (PMC3742406; doi:10.2196/jmir.2579)
Supplement: Supplementary file 5 [file jmir_v15i8e159_app5.pdf]

Eidgenössische Technische Hochschule Zürich
Swiss Federal Institute of Technology Zurich

IBWS Institut für Bewegungswissenschaften und Sport

HIT J 32, Wolfgang-Pauli-Strasse 27, CH-8093 Züric
Eva van het Reve

Technological Familiarity Questionnaire

1. Details to be filled by the interviewer

Name of the interviewed:
Name of the test:
Date:

2. Questions to be answered by the future trainee

2.1. Within the last year, please indicate how much you have used any of the technologies listed below

I don’t know what it is
Not used
Once
Sometimes
Frequently
Automatic teller machine (ATM)

Mobile Telephone

Digital photography
(e.g., camera, camcorder)

Electronic book-reader (e.g., Kindle)

In-car navigation
system (e.g., GPS, OnStar)

In-store automated
kiosk (e.g.,self-checkout)

Eidgenössische Technische Hochschule Zürich
Swiss Federal Institute of Technology Zurich

IBWS Institut für Bewegungswissenschaften

und Sport

HIT J 32, Wolfgang-Pauli-Strasse 27, CH-8093

Züric

Eva van het Reve

MP3/iPod music
player

Recording and
playback device (e.g., CD, DVD, VCR, DVR)

2.2. Have you ever used a computer (i.e., desktop, notebook, or iPad)?

Yes. Which? □ Desktop □ Notebook □ Tablet

No

2.3. How often do you use the computer each week?

Less than 1 hour/week

Between 1-5 hours/week

More than 5, but less than 10 hours/week

10 or more hours/week

2.4. How long have you been using the computer?

Less than 6 months

Between 6 months and 1 year

More than 1 year, but less than 5 years

5 or more years

2.5. Have you ever used the Internet?

Yes

No

2.6. How often do you use the Internet each week?

Less than 1 hour/week

Between 1-5 hours/week

More than 5, but less than 10 hours/week

10 or more hours/week

2.7. How long have you been using the Internet?

Less than 6 months

Between 6 months and 1 year

More than 1 year, but less than 5 years

5 or more years
