# Supplementary material for: Tablet-Based Strength-Balance Training to Motivate and Improve Adherence to Exercise in Independently Living Older People: A Phase II Preclinical Exploratory Trial
Source: J Med Internet Res. 2013 Aug 12;15(8):e159. doi: 10.2196/jmir.2579 (PMC3742406; doi:10.2196/jmir.2579)
Supplement: Supplementary file 6 [file jmir_v15i8e159_app6.pdf]

Eidgenössische Technische Hochschule Zürich
Swiss Federal Institute of Technology Zurich

IBWS Institut für Bewegungswissenschaften und Sport
HIT J 32, Wolfgang-Pauli-Strasse 27, CH-8093 Zürich
Eva van het Reve

Health questionnaire.

Study. - Code: ______________________________

1. **General Questions**

Name: ______________________________

Age: ______

Gender
☐ female ☐ male

Educational background
☐ primary school ☐ vocational education ☐ Secondary school ☐ University/College

Your general Profession was
☐ in sitting position ☐ moderately physical activity ☐ heavily physical activity ☐ hard work

2. **Health Questions**

How do you estimate your health at the moment?
☐ very good ☐ good ☐ middle ☐ bad ☐ I don't know

How do you estimate your health compared to people with the same age?
☐ worse ☐ equal ☐ better ☐ I don't know

How do you estimate your balance skills at the moment?
☐ very good ☐ good ☐ middle ☐ bad ☐ I don't know

Did your general practitioner find one of the following problems?

Diabetes mellitus	☐ yes	☐ no	☐ I don't know
Neuronal Impairment through Diabetes (Polyneuropathy)	☐ yes	☐ no	☐ I don't know
Hypertension	☐ yes	☐ no	☐ I don't know
Cardiac insufficiency	☐ yes	☐ no	☐ I don't know
Hearth attack	☐ yes	☐ no	☐ I don't know
Stroke	☐ yes	☐ no	☐ I don't know
Cancer	☐ yes	☐ no	☐ I don't know
Respiratory disease	☐ yes	☐ no	☐ I don't know
Gastro-Intestinal disease	☐ yes	☐ no	☐ I don't know
Which one? ______________________			
Joint disease (Rheumatism, Arthrosis, Gout)	☐ yes	☐ no	☐ I don't know
Osteoporosis	☐ yes	☐ no	☐ I don't know
Eye disease	☐ yes	☐ no	☐ I don't know
Which one? ______________________			

Which medicine do you take regularly?
(Type, Doses, Frequency, e.g. ASS 100, daily, 1 Pill)

____
____
____

How often do you feel pain?
☐ no ☐ not every day ☐ daily

How intensive do you feel the pain?
☐ lightly ☐ moderately ☐ sometimes unbearable pain

Where do you feel pain? (Location)

IBWS Institut für Bewegungswissenschaften und Sport
HIT J 32, Wolfgang-Pauli-Strasse 27, CH-8093 Zürich
Eva van het Reve

☐ Back pain
☐ Bone pain
☐ Chest pain during normal activity
☐ Head pain
☐ Hip pain
☐ Sore pain
☐ Joint pain (other than hip)
☐ Muscle pain
☐ Stomach
☐ others: ____

Do you suffer from:

Hearing problems:	☐ yes	☐ no
Vision problems:	☐ yes	☐ no
Dizziness:	☐ yes	☐ no
Walking problems:	☐ yes	☐ no

3. **Flexibility Questions**

Do you suffer from leg pain limiting your flexibility?

Foot sores	☐ yes, always	☐ yes, sometimes	☐ no	☐ I don't know
Varicose veins	☐ yes, always	☐ yes, sometimes	☐ no	☐ I don't know
Arthrosis	☐ yes, always	☐ yes, sometimes	☐ no	☐ I don't know
Knee problems	☐ yes, always	☐ yes, sometimes	☐ no	☐ I don't know
Hip problems	☐ yes, always	☐ yes, sometimes	☐ no	☐ I don't know

Others: ____

Do you need a walking aid?

☐ no ☐ Crane ☐ Walking frame

How often did you fall during the last six months?

☐ 0 ☐ 1 ☐ >1

How often in a week and how much time do you spend doing the following recreational activities?

Go for a walk: ____
Gymnastics: ____
Others: ____

Did you do some sports early in life?

☐ yes ☐ no Why did you stop? ______________________________

Have you ever perform a Strength training plan?

☐ yes ☐ no Why did you stop? ______________________________

4. **Question to the intervention**

What do you expect from the exercise program? Aims?

____
____

Do you have any fears?

____
____

IBWS Institut für Bewegungswissenschaften und Sport
HIT J 32, Wolfgang-Pauli-Strasse 27, CH-8093 Zürich
Eva van het Reve
